# Development of a new approach for targeted gene editing in primordial germ cells using TALENs in *Xenopus*

**DOI:** 10.1242/bio.201410926

**Published:** 2015-02-06

**Authors:** Keisuke Nakajima, Yoshio Yaoita

**Affiliations:** Division of Embryology and Genetics, Institute for Amphibian Biology, Graduate School of Science, Hiroshima University, Higashihiroshima 739-8526, Japan

**Keywords:** Primordial germ cells, TALENs, Genomic editing, Targeted gene knockout, *Xenopus tropicalis*

## Abstract

A gene of interest can be efficiently modified using transcription activator-like effector nucleases (TALENs) (Christian et al., 2010;Li et al., 2011). However, if a target gene is essential for development, growth and fertility, use of TALENs with high mutagenic activity in F0 frogs could result in developmental disorders or sterility, which would reduce the number of F1 progeny and make F1 phenotypical analysis difficult. We used the 3′ untranslated region of *DEADSouth* gene (DS-3′) of *Xenopus tropicalis* to solve this problem, because the addition of the DS-3′ to mRNA is known to induce primordial germ cell (PGC)-specific expression and reduce the stability in somatic cells of mRNA in *Xenopus laevis*. At first, we inserted the *X. tropicalis* DS-3′ downstream of the EGFP termination codon and confirmed that the EGFP expression was specifically detected in PGCs for three weeks. Therefore, we inserted the DS-3′ downstream of the termination codon of the TALEN coding sequence. The *tyrosinase* gene was selected as the target gene for TALEN because the bi-allelic mutation of this gene is easily discernible by the albino phenotype. When fertilized eggs were microinjected with TALEN mRNAs fused to the DS-3′, their sperm and oocytes had a high rate (84–100%) of target-gene modification in contrast to the lower rate (0–45%) of nucleotide alteration observed in somatic cells.

## Introduction

Targeted gene disruption is becoming a common, facile and essential method for demonstrating the function of a specific gene and is currently performed using transcription activator-like effector nucleases (TALENs) (Cermak et al., 2011; Christian et al., 2010; Li et al., 2011) or the CRISPR/Cas (clustered regularly interspaced short palindromic repeats/CRISPR-associated proteins) system (Jinek et al., 2012). TALENs are fusion proteins, consisting of a nuclear localization signal, a target DNA-binding domain and the nuclease domain of FokI, that enter the nucleus and recognize 15–24 nucleotides of both the left and right halves of the target sequence to form a dimer in the nuclease domain and catalyze double-strand breaks between the two halves of the target sequence. In the CRISPR/Cas system, ∼20 nucleotides of the target sequence are complementary and bind to the 5′ end of a single synthetic guide RNA, which recruits Cas9 to generate double-strand breaks near the target site. The double strand cleavages formed by these tools are often repaired by non-homologous end-joining, which is an error-prone repair mechanism, resulting in nucleotide deletion and/or insertion.

When gene-modified frogs are generated using the TALEN method or CRISPR/Cas system, different F0 frogs have different mosaic patterns of target gene modification (Blitz et al., 2013; Guo et al., 2014; Ishibashi et al., 2012; Nakajima et al., 2012; Nakayama et al., 2013; Suzuki et al., 2013), and different groups of cells in a single F0 frog possess different mutations at the target locus. Some of the cells in the F0 frog may maintain their functions because they contain an in-frame mutation or lack a mutation, whereas other cells may lose the expression of the target gene because of premature translational termination or nonsense-mediated mRNA decay caused by an out-of-frame mutation. Additionally, some cells may lose the function of the target-gene product because of changes in functionally essential amino acids caused by the insertion and deletion of nucleotides. It is impossible to determine the types of mutation that occur in every cell of the body of an F0 animal, and F0 animals are not suitable for phenotypical analysis to elucidate the function of a gene of interest. It is important to obtain an F1 generation with a bi-allelic null mutation of the target locus to analyze the precise function of a gene because an F1 frog is composed of cells with the common bi-allelic mutation of the target gene, and these mutations can be elucidated. However, when the function of the target gene is indispensable to viability and reproduction, F0 frogs injected with highly active TALEN mRNAs cannot grow to sexual maturity or become fertile adults because of developmental abnormalities and growth arrest. In contrast, if less active TALEN mRNAs are used, it is more difficult to obtain offspring with the bi-allelic mutation. One method of overcoming this dilemma is germ cell-specific disruption of a target gene without somatic cell mutation.

The germ plasm is a cytoplasmic region of the oocyte containing germ cell lineage determinants, including unique mRNAs, proteins, and granules and has been observed in *Drosophila, Xenopus* and *Caenorhabditis elegans* (Ikenishi et al., 1986; Illmensee and Mahowald, 1974; Okada et al., 1974). *DEADSouth* mRNA is localized in the germ plasm of *Xenopus laevis* oocytes (MacArthur et al., 2000; Mosquera et al., 1993) and encodes a putative RNA helicase, a member of the DEAD-box protein family. Primordial germ cells (PGCs) can be visualized in living *X. laevis* embryos by injecting mRNA encoding the coding region of a fluorescent protein and the 3′ untranslated region of *DEADSouth* gene (DS-3′) of *X. laevis* into the vegetal pole of fertilized eggs (Kataoka et al., 2006). The present study was undertaken to test the hypothesis that adding the DS-3′ to TALEN mRNAs may direct the PGC-specific expression of TALENs.

In this study, we succeeded in preferentially editing the genome of the germ cells by injecting TALEN mRNAs fused to the DS-3′ into *Xenopus* embryos.

## Materials and Methods

### Animals

The Ivory Coast line of *X. tropicalis* was provided by the Institute for Amphibian Biology (Graduate School of Science, Hiroshima University) through the National Bio-Resource Project of the MEXT, Japan. The frogs were maintained at 24°C. For the experiments, we used albino *X. tropicalis* frogs that we generated (Nakajima et al., 2012). The male and female frogs were injected with 200 U of human chorionic gonadotropin (ASKA, Tokyo, Japan) dissolved in 0.45% NaCl. The eggs were manually removed from the adult females by squeezing 4–5 hours after the injection. A testis was dissected from a male 2–3 hours after the injection and was suspended in 1 ml of 1×MBS (88 mM NaCl/1 mM KCl/1 mM MgSO_4_/5 mM HEPES (pH 7.8)/2.5 mM NaHCO_3_) containing 0.1% BSA. The testis suspension (100–300 µl) was placed on the eggs, mixed, and allowed to settle in 0.1 × MMR [MMR; 100 mM NaCl/2 mM KCl/2 mM CaCl_2_/1 mM MgCl_2_/5 mM HEPES (pH 7.4)] at 22°C for 8 minutes; then, the fertilized eggs were dejellied using 3% L-cysteine (Sigma) in 0.1×MBS. All of the animals were maintained and used in accordance with the guidelines established by Hiroshima University for the care and use of experimental animals.

### Construction of the reporter and the TALENs

A DNA fragment containing the *X. tropicalis* DS-3′ was amplified using *TaKaRa LA Taq* Hot Start Version (TaKaRa) and the primers XhoXtDS5 (5′-GGCTCGAGTAGGTGTGGCAGCACAA-3′) and XtDS3*Bam*HI (5′-GGGGATCCGAATTTCCCTAAATTGTCTTTACAAAG-3′) in a three-step protocol (94°C for 60 seconds; 35 cycles of 98°C for 10 seconds, 55°C for 30 seconds, and 72°C for 30 seconds; and 72°C for 10 minutes). The product was digested using *Xho*I and *Bam*HI and then ligated into pEGFP-C3 (Clontech). The presence of the DS-3′ was confirmed by DNA sequencing. This construct was designated pEGFP-DS. The DS-3′ fragment was inserted into the *Xba*I site of pCS2-Flag-TALEN-ELD/KKR (Lei et al., 2012) using the In-Fusion Advantage PCR cloning kit to obtain pTALEN-ELD/KKR-DS. The DNA-binding domains were designed to target the first exon of the *tyrosinase* gene (Tyr-B) (Nakajima et al., 2013). TALEN repeats were assembled as previously described (Cermak et al., 2011), with minor modifications (Nakajima et al., 2013), and were inserted into pTALEN-ELD-DS and pTALEN-KKR-DS to generate the Tyr-TALEN-DS expression constructs. The target sequences of Tyr-TALEN-ELD and -KKR were 5′-GGCCCTCAGTTTCCAT-3′ and 5′-GGCCAGTTCTCTCTAT-3′, respectively.

### RNA microinjection

The EGFP-DS DNA fragment was amplified via PCR using the primers T3-pCMV (5′-CGAAATTAACCCTCACTAAAGGGAGGTCTATATAAGCAGAG-3′) and EGFPC3-polyA (5′-TTTTTTTTTTTTTTTTTTTTTTTTTTCCACAACTAGAATGCAGTG-3′). mRNA was transcribed from the EGFP-DS DNA fragment and *Xba*I-digested Tyr-TALEN-DS expression constructs *in vitro* using the mMESSAGE mMACHINE kits (Ambion). Each Tyr-TALEN-DS mRNA (4 nl; 2 or 0.2 ng/µl) and EGFP-DS mRNA (4 nl; 25 ng/µl) dissolved in nuclease-free water (Ambion) was injected into the cortical region of the vegetal pole of fertilized *X. tropicalis* eggs suspended in 6% Ficoll PM 400 (Sigma)/0.1×MMR/0.1% BSA (supplementary material Movie 1). Fluorescence was used to identify embryos that had been successfully injected and thus contained EGFP-positive PGCs (Kataoka et al., 2006). The embryos were raised at 22–24°C in 0.1×MMR containing 0.1% BSA and 50 µg/ml gentamycin. Tadpoles were anesthetized, and the EGFP expression in PGC was photographed under a fluorescent dissecting microscope (MZ FLIII, Leica) equipped with a color CCD camera DP70 (Olympus).

### DNA extraction

The phenotypes of the F1 tadpoles were assessed seven to ten days after fertilization (at stages 47–48). The tip of the tail of each tadpole was homogenized in 90 µl of 50 mM NaOH and incubated for 10 minutes at 95°C. The homogenate was mixed with 10 µl of 1 M Tris-Cl (pH 8.0) and centrifuged at 1500×g for 10 minutes at room temperature. The supernatant was extracted using phenol and chloroform. An organ or tissue was mixed with 80 µl of 50 mM Tris-HCl (pH 7.4) containing 1 mM CaCl_2_. After the addition of 5 µl of 10% SDS and 3 µl of 10 mg/ml proteinase K, the mixture was incubated at 65°C for a few hours. Then, 2 µl of 0.1 M PMSF and 0.5 µl of 20 mg/ml RNase A were added, and the mixture was incubated at 37°C for 10 minutes. The genomic DNA was purified using Wizard PCR Preps DNA Purification Resin (Promega).

### Mutation analysis

DNA fragments containing the target site were amplified using the EmeraldAmp MAX PCR Master Mix (TaKaRa) and the primers Tyr-F1 (5′-TCACAGAAAGGGTTAAGGGGAAG-3′) and Tyr-R1 (5′-GCACCCCTACAACAGCCTTC-3′) for 25 cycles (95°C, 30 seconds; 58°C, 30 seconds; 72°C, 120 seconds). The second round of PCR was performed using Tyr-F2 (5′-GTGAGGAGCAGCATGGAA-3′) and Tyr-R2 (5′-CTGCCATGAAGCGAAGAAGGATG-3′) for 20 cycles (95°C, 30 seconds; 58°C, 30 seconds; 72°C, 60 seconds) (Fig. 3A). The PCR products were subcloned into the pGEM-T Easy vector (Promega), and the nucleotide sequences were subsequently determined. When we identified *tyrosinase* mutations derived only from albino mates (Fig. 3F), PCR was conducted using Tyr-F1, Tyr-R1 and TaKaRa Ex Taq Hot Start Version (TaKaRa) for 35 cycles (95°C, 30 seconds; 58°C, 30 seconds; 72°C, 120 seconds), and DNA fragments with 620-bp and 952-bp deletions were cloned. When we did not observe two different alleles in individual offspring of the m1 and f1 frogs via a first round of PCR using the Tyr-FI and -R1 primers and a second round of PCR using the Tyr-F2 and -R2 primers (Fig. 4B), the second round of PCR was exchanged for a protocol that used the Tyr-F1 and Tyr-R2 primers for 20 cycles (95°C, 30 seconds; 58°C, 30 seconds; 72°C, 120 seconds), and a fragment with a 339-bp deletion was cloned. Alternatively, the target DNA was amplified using genomic DNA and the Tyr-F3 (5′-TGCGACGAAATAATACCCGCA-3′) and Tyr-R1 primers for 35 cycles (95°C, 30 seconds; 58°C, 30 seconds; 72°C, 180 seconds) using TaKaRa Ex Taq Hot Start Version (TaKaRa). A DNA fragment with a 615-bp deletion was obtained. The PCR products were subcloned and their nucleotide sequences were determined.

## Results

**Fig. 1.**
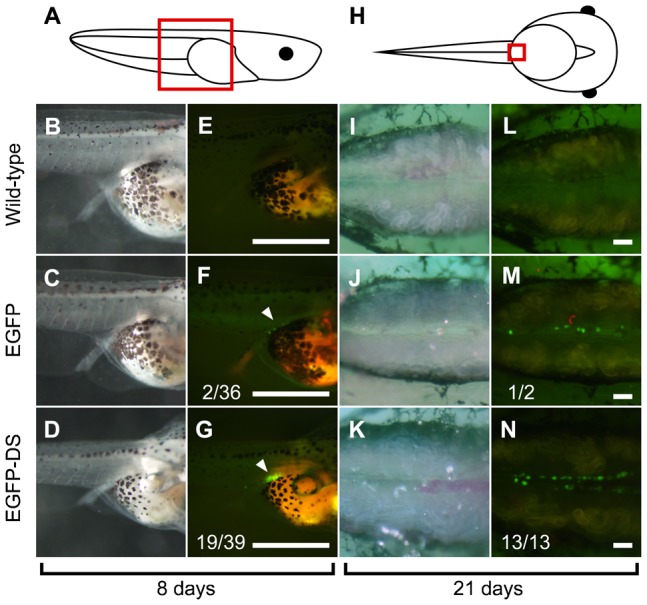
Expression of EGFP in *X. tropicalis* tadpoles injected with EGFP-DS mRNA. (A,H) A schematic representation of a lateral view of an 8-day-old tadpole (A) and a ventral view of a 21-day-old tadpole (H). (B–G) Higher-magnification view of A. (I–N) Higher-magnification view of H. (B,E,I,L) Images of wild-type tadpoles. (C,F,J,M) Images of tadpoles injected with EGFP mRNA. (D,G,K,N) Images of tadpoles injected with EGFP-DS mRNA. (B–D) Brightfield images of live 8-day-old tadpoles. (E–G) EGFP expression in B, C and D, respectively. (I–K) Brightfield images of the mesonephros of 21-day-old tadpoles. (L–N) EGFP expression in I,J and K, respectively. F and G show the ratio of the number of EGFP-positive tadpoles on the eighth day to the number of mRNA-injected tadpoles. M and N show the ratio of the number of EGFP-positive tadpoles on the 21st day to the number of EGFP-positive tadpoles on the eighth day. The white arrowheads indicate PGCs. Scale bars = 1 mm in (E–G) and 0.1 mm in (L–N).

We hypothesized that injecting TALEN mRNAs fused to the DS-3′ would result in PGC-specific TALEN expression. To test this hypothesis, we constructed an EGFP plasmid, pEGFP-DS, in which the *X. tropicalis* DS-3′ was positioned downstream of the termination codon of the EGFP fluorescent protein. EGFP and EGFP-DS mRNAs were transcribed from pEGFP-C3 and pEGFP-DS, respectively, and injected vegetally into fertilized *X. tropicalis* eggs. EGFP fluorescence was frequently observed in the PGCs in the mesentery of eight-day-old tadpoles (19 EGFP-positive tadpoles on the eighth day out of 39 tadpoles injected with EGFP-DS mRNA) and in the genital ridges of 21-day-old tadpoles (13 EGFP-positive tadpoles on the 21st day out of 13 EGFP-positive tadpoles on the eighth day). In contrast, when EGFP mRNA was injected, EGFP expression was rarely detected in the PGCs of eight-day-old tadpoles (two EGFP-positive tadpoles on the eighth day out of 36 tadpoles injected with EGFP mRNA) and was detected in the PGCs of only one 21-day-old tadpole (one EGFP-positive tadpole on the 21st day out of two EGFP-positive tadpoles on the eighth day) (Fig. 1). These results showed that the EGFP protein that was translated from EGFP-DS mRNA was stable in a PGC-specific manner for three weeks, and this expression pattern could be ascribed to the presence of DS-3′ in the injected mRNA.

**Fig. 2.**
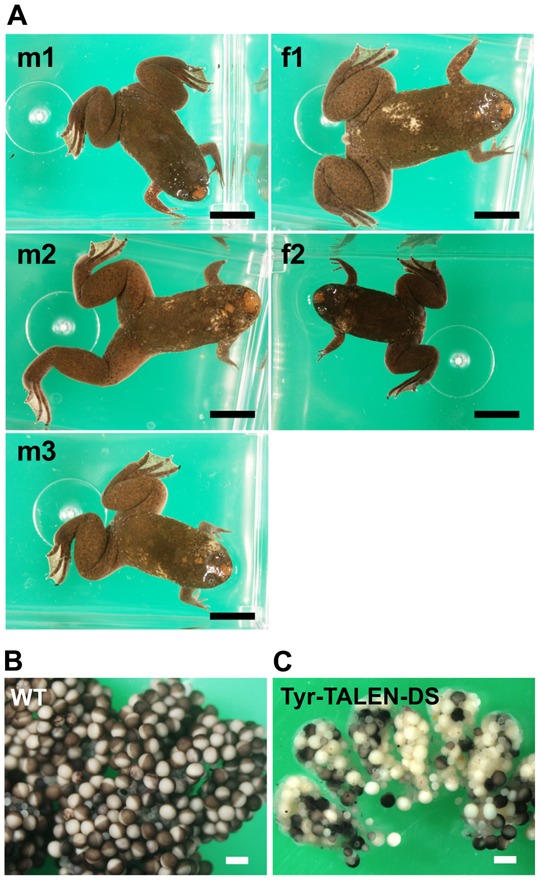
Phenotype of F0 frogs. (A) Photographs of F0 frogs. Tyr-TALEN-DS mRNAs were co-injected with EGFP-DS mRNA at the vegetal pole of fertilized eggs. The frogs were allowed to reach sexual maturity. Scale bars = 1 cm. (B) Ovary of a wild-type one-year-old frog. (C) Ovary of a one-year-old F0 frog that was injected with Tyr-TALEN-DS mRNAs. (B,C) Scale bars = 1 mm.

The mRNAs that were synthesized using the Tyr-TALEN-DS expression constructs were designed to target the *X. tropicalis tyrosinase* gene; these constructs contained the obligatory heterodimeric nuclease domains (Lei et al., 2012; Nakajima and Yaoita, 2013) and the DS-3′ of *X. tropicalis*. Tyrosinase is essential for melanin synthesis, and the bi-allelic disruption of the *tyrosinase* gene leads to the albino phenotype, which is easily discernible (Blitz et al., 2013; Guo et al., 2014; Ishibashi et al., 2012; Nakajima et al., 2012; Nakayama et al., 2013). Although fertilized *X. tropicalis* eggs are generally injected with 400 pg of TALEN mRNAs, in this study, we injected 16 pg or 1.6 pg of Tyr-TALEN-DS mRNAs because the translated proteins were expected to be expressed in the PGCs for more than three weeks and because injecting a smaller amount of exogenous mRNA is more conducive to embryonic viability and development. The TALEN mRNAs were co-injected with EGFP-DS mRNA to confirm that the injected EGFP-DS mRNA was translated in the migratory PGCs. The tadpoles that contained EGFP-positive PGCs two days after injection developed into sexually mature adult frogs that had a few tiny patches of depigmentation in their skin (Fig. 2A).

**Fig. 3.**
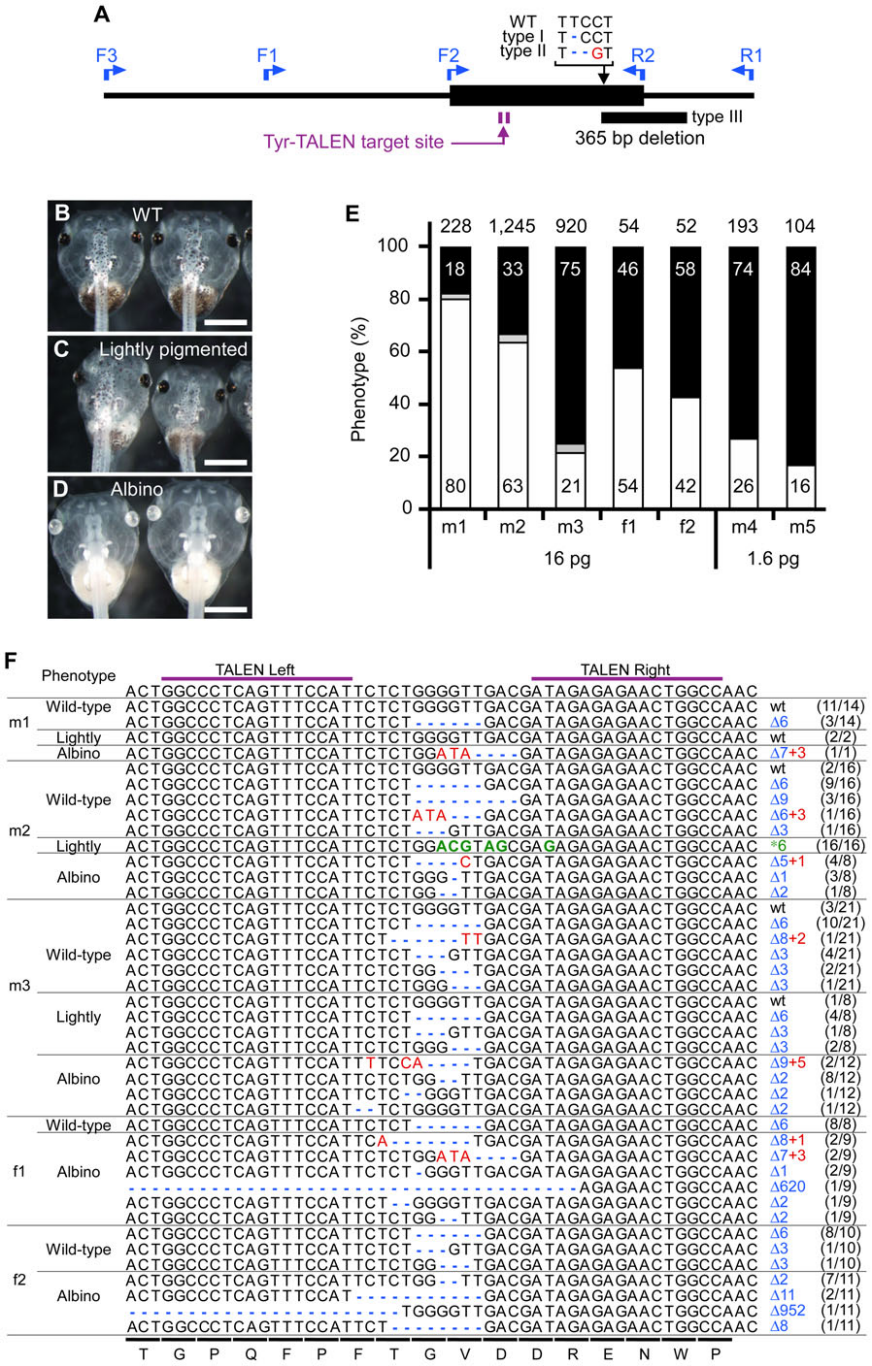
Analysis of individual offspring obtained from crosses between F0 and albino frogs. (A) Schematic representation showing both the gene mutations of the albino mates used in the crosses and the locations of primers used in the mutation analysis. The bold horizontal line indicates the DNA region from the initiation codon to the end of the first exon. The mutated sequences of the albino mates are shown as type I, type II and type III with the wild-type sequence. The inserted and deleted nucleotides are indicated with a red character and blue dashes, respectively. The locations of the mutations and the 365-bp deletion in the albino mates are indicated by a black arrow and rectangle, respectively. The Tyr-TALEN target site and the direction of the primers are indicated by a purple arrow and blue arrows, respectively. (B–D) Brightfield images of live wild-type (B), lightly pigmented (C) and albino (D) tadpoles. Note that the abdominal region is lightly pigmented in C. Scale bars = 1 mm. (E) Phenotypic analysis of the offspring of F0 and albino frogs. The percentages of wild-type, lightly pigmented and albino F1 tadpoles are indicated by black, gray and white bars, respectively (Table 1). The numbers in the bars are the percentages of tadpoles with the indicated phenotype. The total number of tadpoles is shown at the top of the bars. (F) Genotypic analysis of individual offspring of F0 and albino frogs. The target DNA fragment was amplified using genomic DNA purified from individual wild-type, lightly pigmented and albino F1 tadpoles and was recloned for sequence determination. All of the mating data are shown. Different sequences were bequeathed by F0 frogs to different F1 offspring, which also had the other mutated *tyrosinase* allele inherited from the albino parent. The target sequences derived from the albino mates are not shown. The wild-type target DNA and the amino acid sequences are indicated at the top and bottom of the panel, respectively. A pair of purple bars denotes the TALEN-binding sites. The gaps resulting from a deletion (Δ), the inserted nucleotides (+) and the exchanged nucleotides (*) are indicated with blue dashes, red characters and green characters, respectively. The mutation types are indicated on the right. The ratio of the number of tadpoles with the indicated sequence to the total number of tadpoles with the same phenotype is shown in parentheses on the right. Large deletions of 620 and 952 nucleotides were observed in the offspring derived from the f1 and f2 frogs, respectively, and both of these deletions contained the start codon.

The male F0 frogs were mated to albino females to estimate the frequency of *tyrosinase* gene disruption in the germ cells based on the ratio of albino to non-albino offspring. The allele from a male F0 frog was easily distinguished from the allele from albino female in offspring because the albino mates used in the crosses have two out of three types of mutations that are located far from the targeting site of Tyr-TALEN (Fig. 3A). Albinism was observed in 80%, 63% and 21% of the offspring of albino females crossed to three different males injected with 16 pg mRNA, which were designated as m1, m2 and m3, respectively (Fig. 3B–E); these results imply that the *tyrosinase* gene was mutated in ∼21–80% of the spermatozoa. Some of the resultant tadpoles were lightly pigmented (Fig. 3C). To determine their individual genotypes, the *tyrosinase* gene was cloned and sequenced. In the lightly pigmented offspring, almost all of the *tyrosinase* alleles that were expected to have been derived from the m2 and m3 males had nucleotide deletions or substitutions, but maintained an in-frame coding region (Table 1), suggesting that these in-frame mutations reduced the activity of the enzyme but did not result in complete gene inactivation. Furthermore, genotypic analysis of F1 wild-type offspring revealed that 21%, 88% and 86% of the alleles derived from the m1, m2 and m3 males, respectively, were modified and were still in-frame, implying that these in-frame mutations had little effect on the activity of the enzyme. Based on these data, we estimated the mutation hit rates of the *tyrosinase* gene to be 84%, 96% and 89% in the spermatozoa of the m1, m2 and m3 males, respectively (Table 1).

**Table 1.**
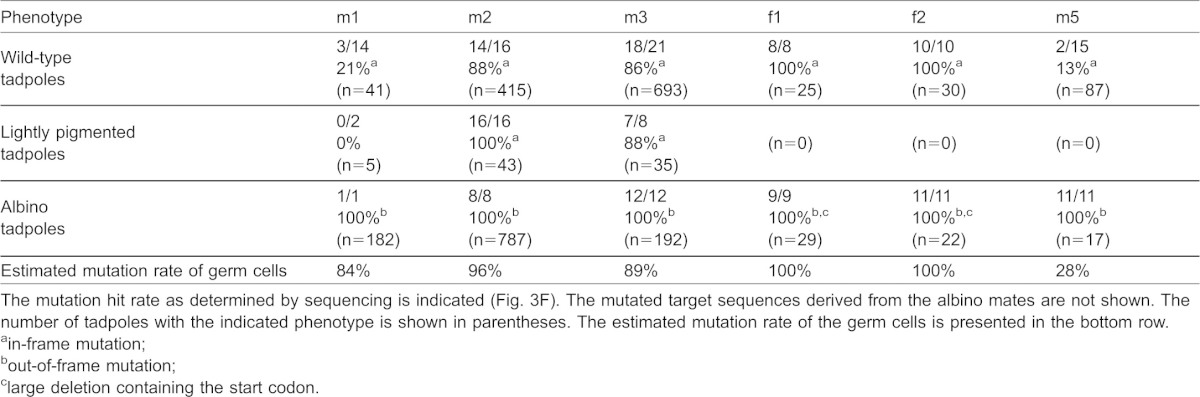
Genotypic analysis of individual offspring of F0 and albino frogs

Albino tadpoles comprised 26% and 16% of the offspring derived from crosses between albino females and 1.6-pg-mRNA-injected m4 and m5 males, respectively, and the gene modification rate in the m5 spermatozoa was estimated to be 28% using genotypic analysis (Table 1). When the amount of injected TALEN mRNAs was reduced to one-tenth of the original amount, the frequency of the albino phenotype and the mutation hit rate both decreased to approximately one-third of their prior levels.

Conversely, the albino phenotype appeared in 54% and 42% of offspring resulting from crosses between albino males and 16 pg-mRNA-injected f1 and f2 female frogs, respectively, and all of the examined *tyrosinase* alleles derived from f1 and f2 oocytes were found to be modified (Table 1). F0 female frogs had many albino oocytes in their ovary compared to the wild-type ovary (Fig. 2B,C).

**Fig. 4.**
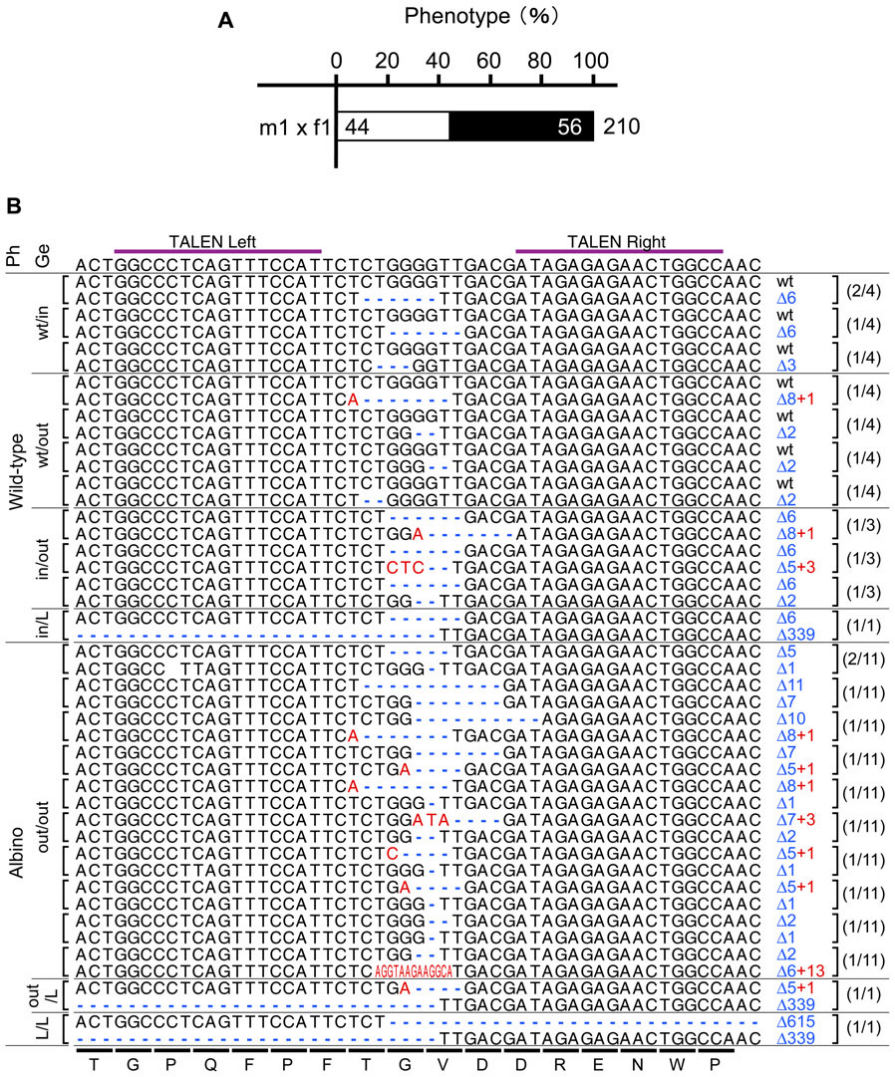
Analysis of individual offspring of F0 frogs. (A) Phenotypic analysis of the offspring obtained by mating two F0 frogs, m1 and f1. The percentages of wild-type and albino F1 tadpoles are indicated by black and white bars, respectively, and the values are denoted in the bars. The total number of tadpoles is shown on the right. (B) Genotypic analysis of individual offspring obtained from mating the m1 and f1 frogs. The target DNA fragment was amplified using genomic DNA that was purified from individual F1 tadpoles and was recloned for sequence determination. The phenotype (Ph) and genotype (Ge) of F1 tadpoles are indicated on the left. Each F1 tadpole had a pair of sequence lines as shown in brackets. wt, wild-type target sequence; in, in-frame mutation; out, out-of-frame mutation; L, large deletion containing the start codon or the exon-intron boundary. The ratio of the number of tadpoles with a pair of the indicated sequences to the total number of tadpoles with the same phenotype and genotype is shown in parentheses on the right. The alignment is labeled as described in the legend of Fig. 3F.

Mating of two F0 frogs, m1 and f1, resulted in 44% albino offspring, which was consistent with the frequency estimated from the ratios of albino to non-albino offspring obtained by mating m1 or f1 with albino partners (Fig. 3E and Fig. 4A). This result shows that it is possible to efficiently generate an F1 bi-allelic gene-knockout using our method (Table 2; Fig. 4).

**Table 2.**
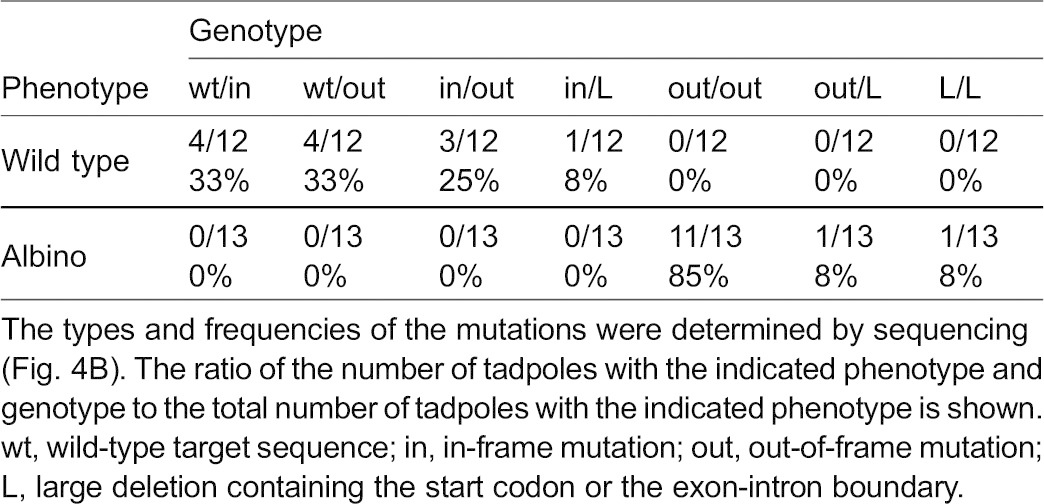
Results of the genotypic analysis of individual offspring of F0, m1 and f1 frogs

**Fig. 5.**
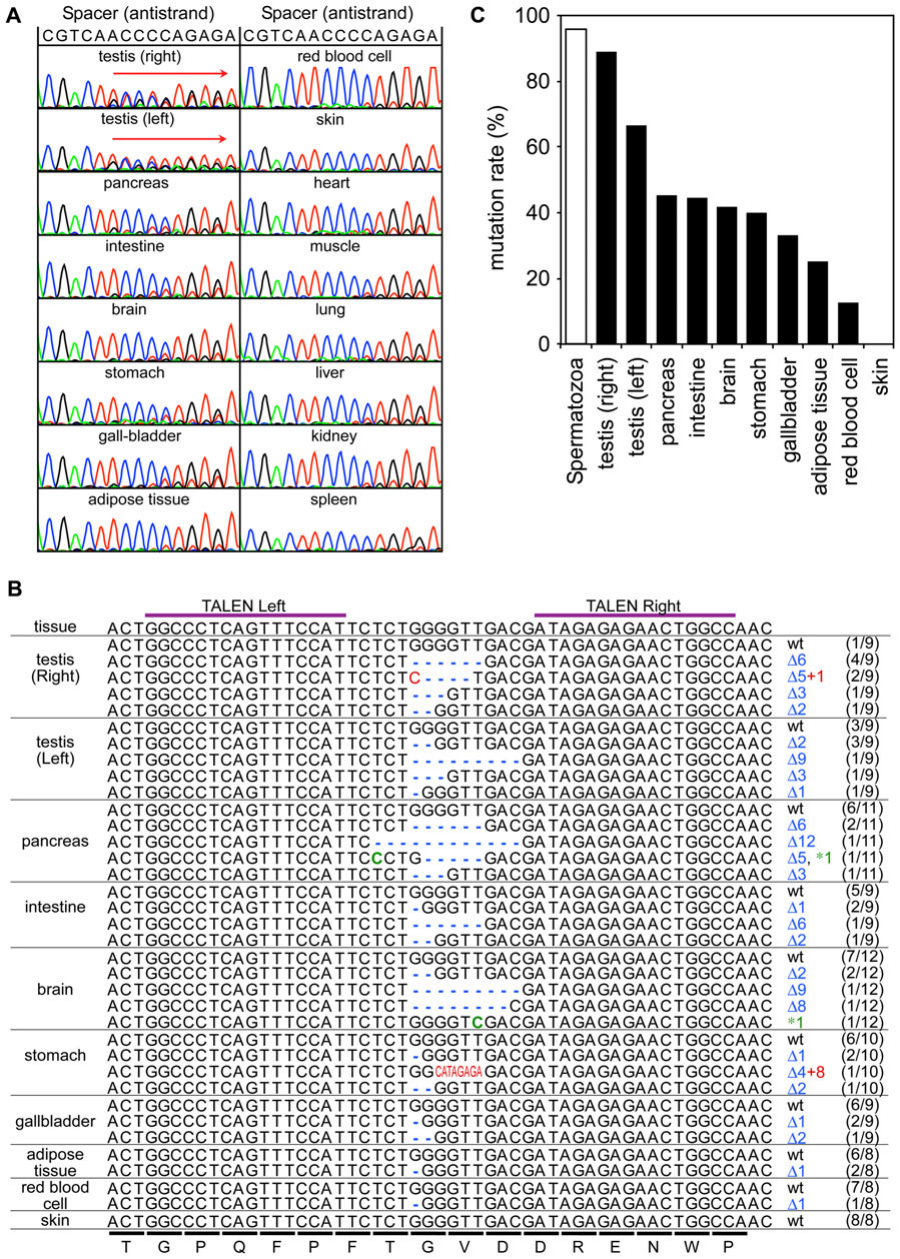
Preferential mutagenesis in the testes of the m2 frog. (A) Simple direct sequencing of the PCR-amplified targeted genome region (DSP assay) using genomic DNA obtained from several organs and tissues of the Tyr-TALEN-DS-mRNA-injected m2 frog. The spacer sequence located between the Tyr-TALEN binding sites is shown at the top of the panel. The ambiguous sequence in the right and left testes begins with “A” (a red wave) and extends to the right, as shown by the red arrows, suggesting that these amplicons are mixtures of heterogeneous fragments with different mutations. (B) Mutated target sequences in organs and tissues of the m2 frog. The target DNA fragment was amplified using genomic DNA that was purified from organs and tissues and subcloned. The sequences of eight to 12 clones per organ or tissue were determined. The ratio of the number of the indicated sequence to the total number of sequences in an organ or tissue is shown in parentheses on the right. The alignment is labeled as described in the legend of Fig. 3F. (C) Mutation hit rate in several organs and tissues of m2. The mutation hit rate in an organ or tissue is indicated by a black bar, whereas the estimated mutation rate in the m2 spermatozoa is indicated by a white bar.

To confirm that the targeted gene mutation was induced specifically in germ cells by the Tyr-TALEN-DS mRNAs, the genomic DNA was extracted from several organs and tissues of the m2 frog and subjected to simple direct sequencing of the PCR-amplified targeted genome region (DSP assay) (Nakayama et al., 2013). A sequence comparison showed that the *tyrosinase* gene was modified in the genomic DNA of the right and left testis but was not often modified in the genomic DNA of other organs (Fig. 5A). To examine the organ specificity of the mutation of this gene, the *tyrosinase* gene was cloned from the genomic DNA of various organs and tissues and then sequenced (Fig. 5B,C). The mutation hit rate was higher in the testes (67–89%) than in the other organs (0–45%). No mutation was detected in randomly selected eight clones containing the *tyrosinase* gene derived from genomic DNA of m2 frog skin. This may be because the prospective epidermis is located far away from the cortical region of the vegetal pole, which is the mRNA injection site. It is possible that the m2 sperm were derived mainly from the right testis during mating with albino females because the spermatozoa and the right testis had higher mutation rates (96% and 89%, respectively) than the left testis (67%). Alternatively, the testes may include many non-germ cells containing the wild-type *tyrosinase* gene even though nearly all of the germ cells have mutations in this gene.

## Discussion

We report a simple and efficient method of preferentially editing the genome of germ cells using TALEN, which may enable the generation of F1 frogs with a bi-allelic target-gene mutation through mating of healthy and fertile F0 frogs even when the gene of interest is necessary for viability, normal development, homeostasis or reproduction. In the m2 male, the hit rate for *tyrosinase* gene mutation was 96% in spermatozoa and ∼40% in the pancreas, intestine, brain and stomach. Therefore, the bi-allelic gene modification rate may have been 16% in pancreatic cells and cells of other organs. Even if the 16% of the cells with the bi-allelic target-gene mutation did not survive within the pancreas or other organs, the remaining cells with one or two copies of the wild-type gene may compensate for the dead cells. However, when the target gene is necessary for the survival of germ cells or the fertilization itself, F1 offspring cannot be obtained by our method. We have not performed the side-by-side experiment to examine the ratios of germ line transmission in TALEN-DS-mRNA-injected embryos to those in TALEN-mRNA-injected embryos. However, the result in embryos injected with EGFP-DS and EGFP mRNAs strongly suggests that the efficiency of germ line transmission is enhanced by adding DS-3′ to TALEN mRNAs.

The germ line of the F0 animals was highly mosaic, as demonstrated by the genotyping of F1 animals generated by mating two F0 frogs, m1 and f1. Twenty different types of mutation were observed in the F1 offspring. Furthermore, the genetic mosaicism of the F0 animals is consistent with the results of many studies in which zinc-finger nuclease, TALEN and CRISPR/Cas systems were used (Blitz et al., 2013; Guo et al., 2014; Ishibashi et al., 2012; Nakajima et al., 2012; Nakayama et al., 2013; Suzuki et al., 2013). Although genetically heterogeneous F0 animals show a similar phenotype, this result suggests that knocking out the gene of interest induced this phenotype. Furthermore, if the F0 frogs do not show any phenotype, no conclusions regarding the gene in question can be drawn. However, the necessity and functional redundancy of the gene can be evaluated, when no phenotype is evident in the F1 animals with a bi-allelic out-of-frame mutation.

When fertilized eggs were injected vegetally with mRNA encoding a fluorescent protein fused to the DS-3′ of *X. laevis*, a diffuse signal was observed in the vegetal blastomeres of the blastula embryos, and this signal appeared to be restricted to the PGCs from the mid-blastula transition (MBT) stage onward (Kataoka et al., 2006). The mRNA fused to the DS-3′ was degraded in the somatic cells via miR-427-mediated mRNA clearance after the MBT stage, but not in PGCs (Yamaguchi et al., 2014). Because the TALEN mRNAs fused to the DS-3′ would be translated before the MBT stage, the target gene should be modified in somatic cells using our method, which would impair the germ-cell specificity of the genomic editing. If translation of the TALEN-DS mRNAs could be repressed using anti-sense oligo-deoxynucleotides or RNA for several hours until the MBT stage is reached, the target gene may be protected from degradation in somatic cells.

Two modes of germ-cell specification have been proposed: the inductive (regulative) mode and the predetermined (germ plasm) mode (Bachvarova et al., 2009; Extavour and Akam, 2003). In the inductive mode, cells of the epiblast or the animal cap would not normally form germ cells at the late blastula to the early gastrula stages, but they can be induced to form these cells in the presence of the appropriate tissue or growth factors. In the predetermined mode, germ cells are specified before gastrulation by the germ plasm, the maternally inherited determinants present in the egg. This mode is used by nematodes, flies, tunicates, teleosts, frogs and birds for PGC specification. Our method should be applicable for genomic editing in animals that employ the predetermined mode but not in those using the inductive mode because the germ plasm contains localized maternal mRNAs. For example, *nanos1* mRNA is one component of zebrafish germ plasm, and its localization is restricted to the PGCs by its 3′UTR, which is similar to the DS-3′ (Köprunner et al., 2001). If zebrafish embryos are injected with TALEN mRNAs fused to the *nanos1* gene 3′UTR, this mRNA should be localized and translated specifically in PGCs, leading to digestion of the target sequence within the genome.

In *Drosophila*, a genomic editing method was recently developed for specifically targeting germline cells; in this system, a single guide RNA-expression construct is injected into the embryos of a transgenic line expressing Cas9 in the germline (Ren et al., 2013). Although the overall heritable mutation rates are high (12.4–74.2%), the germ-cell specificity of genomic editing was not examined, and this system is available only for Cas9-expressing transgenic lines, in contrast to our method, which uses wild-type animals.

Our TALEN system simply and efficiently generated heritable mutant alleles in germ cells with less frequent somatic mutations using only TALEN mRNAs fused to the DS-3′. This method may enable the generation of F1 offspring possessing a bi-allelic mutation of a lethal gene through mating of fertile F0 individuals. Furthermore, this method is applicable to genomic editing in animals that use the predetermined mode of germ-cell specification if the 3′UTR of a germ plasm-specific mRNA, similar to the DS-3′, determines the location of this mRNA in the PGCs.

## Supplementary Material

Supplementary Material
