# Supplementary material for: Development of a new approach for targeted gene editing in primordial germ cells using TALENs in Xenopus
Source: Biol Open. 2015 Feb 6;4(3):259–66. doi: 10.1242/bio.201410926 (PMC4359732; doi:10.1242/bio.201410926)
Supplement: Supplementary Material [file supp_4_3_259__index.html]

Development of a new approach for targeted gene editing in primordial germ cells using TALENs in Xenopus — Supplementary Material 

# Development of a new approach for targeted gene editing in primordial germ cells using TALENs in *Xenopus*

## bio.201410926 Supplementary Material

**Files in this Data Supplement:**

- Supplementary Material - Keisuke Nakajima and Yoshio Yaoita doi: 10.1242/bio.201410926
- Movie 1 - **The injection of TALEN-DS mRNAs into the cortical region of the vegetal pole of fertilized *X. tropicalis* eggs.**
