# Supplementary material for: Development of a new approach for targeted gene editing in primordial germ cells using TALENs in Xenopus
Source: Biol Open. 2015 Feb 6;4(3):259–66. doi: 10.1242/bio.201410926 (PMC4359732; doi:10.1242/bio.201410926)
Supplement: Supplementary Material [file supp_bio.201410926_bio.201410926-s1.pdf]

Biology Open (2015) 000, 1–8 doi:10.1242/bio.201410926

Supplementary Material

Keisuke Nakajima and Yoshio Yaoita doi: 10.1242/bio.201410926

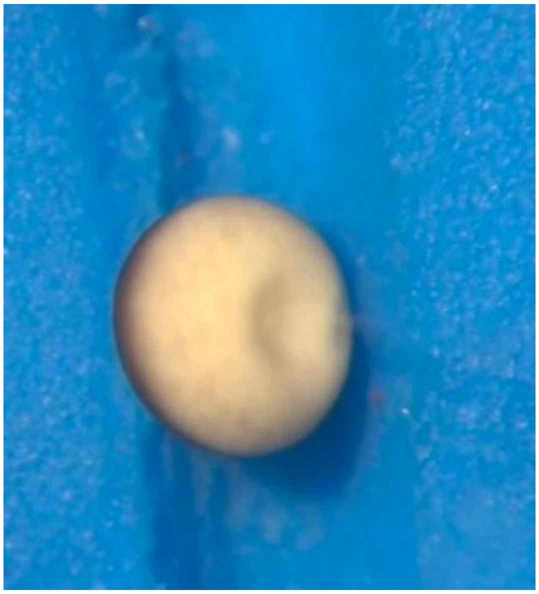

Movie 1. The injection of TALEN-DS mRNAs into the cortical region of the vegetal pole of fertilized *X. tropicalis* eggs.

Biology Open
